# Hydrocyanic Acid Should Be Stricken from the List of Officinal Preparations

**Published:** 1860-10

**Authors:** F. Mahla


### HYDROCYANIC ACID SHOULD BE STRICKEN FROM THE LIST OF OFFICINAL PREPARATIONS.

(From a Paper on Hydrocyanic Acid, by Dr. F. MAHLA.)

Hydrocyanic (prussic) acid is, in the hands of an experienced practitioner, undoubtedly of great therapeutic value. Yet there are serious objections as to the forms in which it is generally prescribed by physicians. Hydrocyanic acid is employed in various forms. The U. S. Pharmacopœa names as officinal preparations not only a diluted hydrocyanic acid, but gives also directions for the preparation of other medicaments which contain this substance. The aqua amygdolarum amararum, the oil of bitter almonds, are pharmaceutical preparations, which are constantly varying in strength in regard to prussic acid. It is well known, that hydrocyanic acid is liable to undergo decomposition, not only if exposed to the direct sunrays, but also if exposed to common daylight. It has been observed that it became brown and turbid even if the bottle was covered with black paint or paper. This shows clearly that great moleoular changes take place readily under certain circumstances, which are out of control. It is obvious that all the other hydrocyanic acid containing preparations, are subject to the same decompositions.

There is still another serious objection to the use of ·those medicaments in medical practice. Hydrocyanic acid is a chemical compound, which is highly volatile. The anhydrous acid boils already at 80° F. As our usual summer temperature is sometimes 90 or even 100 degrees, it may be easily conceived, that the absorbing power of water of that temperature is considerable less than that of water of 30 or 40 degrees. If, therefore, a bottle containing this preparation be opened during the hot days of July or August, and a portion of the contents taken out, it is but natural that a certain quantity of gaseous hydrocyanic acid escapes from its aqueous solution and fills the empty space of the bottle. This of course escapes into the air as soon as the vial is opened once more. It becomes thus weaker and weaker, and the practitioner waits in vain for that effect, which a former dose always used to produce.

It is very frequently the case, that an acid, even if directly from the chemical manufacturer, has not the required strength. According to the U. S. Ph., hydrocyanic acid should contain 2 per cent. of anhydrous acid. In most cases, it is, however, considerably weaker, and becomes the more so the longer it is kept on hand.

Though these facts are well known to every professional chemist, I thought it would be of interest to the practitioner if I could prove their truth by direct experiments. I subjected, therefore, different samples of hydrocyanic acid, of aqua amygd. am. and of ol. amygd. eath. to a quantitative analysis. I made use, for these determinations, of Liebig's method, with a silver solution of known strength, caustic potassa and chloride of sodium, as described in "Mohr's Titrir-book."

| | | | |
|---|---|---|---|
| Acid A. perfectly colorless, top well secured, analyzed 2 months after shipment from the factory,............... | 1.44 | per cent. of anhydrous acid. | |
| Same acid analyzed 14 days afterwards,................ | 1.32 | " | " |
| Acid B. received from the factory 3 months previous, well stoppered,............ | 0.95 | " | " |
| Acid of the same manufac. kept in store for about 5 months, (bottle had been opened during this period),. | 1.02 | " | " |

Essential oil of bitter almonds,.................. 6.30 per. cent. of anhydrous acid.

Aqua amygd. amar., fresh prepared according to the U. S. D.,.....................0.0036 " "

Aqua amygd. am., prepared 6 months ago, but kept in a well closed bottle,........0.0000 " "

It is obvious, that a remedy, the exact strength of which cannot be controlled, is not fit to be used in medical practice. The question is now, in what form shall the physician prescribe hydrocyanic acid? Cyanid of potassium, as it occurs in commerce, is not pure; it contains always cyanate of potassa, besides occasional impurities. The pure cyanid of potassium is very difficult to prepare, very delinquescent, and its solution very prone to decomposition. It is, therefore, a substance not fit to be dispensed by the pharmaceutist.

To avoid these difficulties, Prof. Wohler suggested already some time ago, the use of amygdalin. This principle, which is contained in bitter almonds, is readily decomposed by the combined action of emulsin and water into the essential oil of bitter almonds, prussic acid and sugar The oil of bitter almonds (when deprived of its prussic acid,) is without effect upon the animal system, at least in all doses in which it would be prescribed, and it could be therefore of no objection to have it as an accompanying admixture in a medicament.

Seventeen grains of amygdalin produce, if acted on by emulsin and water, one grain of anhydrous prussic acid.

A teaspoonful of a mixture made according to the following formula, would contain, therefore, a dose of 2 drops of the officinal diluted hydrocyanic acid, viz:

℞. Emuls. amygdal. dulc., (ex. ʒii,) ℥ iv.

Amygdalin pur. pulver., gr. xx. Misce.

